# Platelet transfusion refractoriness within one month post-hematopoietic stem cell transplantation does not impair survival in aplastic anemia patients after engraftment: a propensity score-matched analysis

**DOI:** 10.3389/fimmu.2025.1623004

**Published:** 2025-07-24

**Authors:** Yan Wang, Yuanfeng Zhang, Li Liu, Tingting Zhang, Jiali Sun, Xin Chen, Donglin Yang, Aiming Pang, Rongli Zhang, Qiaoling Ma, Weihua Zhai, Yi He, Jialin Wei, Yigeng Cao, Chen Liang, Erlie Jiang, MingZhe Han, Sizhou Feng

**Affiliations:** ^1^ State Key Laboratory of Experimental Hematology, National Clinical Research Center for Blood Diseases, Institute of Hematology and Blood Diseases Hospital, Chinese Academy of Medical Sciences and Peking Union Medical College, Tianjin, China; ^2^ Department of Hematology, The Affiliated Yantai Yuhuangding Hospital of Qingdao University, Yantai, Shandong, China

**Keywords:** aplastic anemia, transplantation, platelet transfusion refractoriness, propensity score, survival

## Abstract

**Introduction:**

The incidence of platelet transfusion refractoriness (PTR) and its impact on survival outcomes in patients with severe aplastic anemia (SAA) undergoing allogeneic hematopoietic stem cell transplantation (allo-HSCT) remains unclear.

**Methods:**

We investigated the incidence of early PTR (within one month post-allo-HSCT) and its clinical implications in 215 aplastic anemia (AA) patients in a retrospective study.

**Results:**

Among the enrolled patients, 24 (11.7%) developed PTR within the first month post-transplantation. Propensity score matching (PSM) was performed, resulting in 24 PTR cases and 96 matched non-PTR controls, with balanced baseline characteristics. No significant differences were observed between the two groups in bloodstream infections, grade II–IV or III–IV acute graft-versus-host disease (aGVHD), viral infections, or engraftment rates. However, PTR patients required significantly more red blood cell (median: 13.5 units vs. 8 units, *P* = 0.003) and platelet transfusions (median: 10.5 units vs. 5 units, *P* < 0.001) compared to non-PTR patients. The 3-year overall survival (OS) rate was numerically lower in the PTR group (66.7%; 95% CI, 44.3–81.7) than in the non-PTR group (81.2%; 95% CI, 71.1–88.0), although this difference was not statistically significant (*P* = 0.106). Multivariate analysis identified haploidentical donor and patient age as independent risk factors for OS.

**Conclusion:**

Our findings suggest that early PTR occurs at a relatively low frequency (11.7%) in AA patients post-allo-HSCT and may not significantly compromise survival outcomes following successful engraftment.

## 1 Introduction

Platelet transfusion refractoriness (PTR), characterized by inadequate post-transfusion platelet count recovery (1), results from both immunologic and non-immunologic mechanisms. Unlike chemotherapy patients, PTR following allogeneic hematopoietic stem cell transplantation (allo-HSCT) predominantly stems from non-immunologic factors such as infection, fever, and bleeding (2). The clinical impact of post-allo-HSCT PTR remains controversial, with studies reporting conflicting outcomes (3, 4); this discrepancy may reflect variations in PTR onset timing, underlying diseases, and conditioning regimens (4). Of note, recent work by Gao et al. have demonstrated that rabbit anti-thymocyte globulin (rATG) can effectively reverse PTR in severe aplastic anemia (SAA) patients receiving intensive immunosuppressive therapy (5). However, the incidence and survival implications of early PTR, within one month post-allo-HSCT, in aplastic anemia (AA) patients have not been established. To address this knowledge gap, we conducted a retrospective study examining the incidence and clinical consequences of early PTR in AA patients undergoing allo-HSCT.

## 2 Methods

### 2.1 Patients

From 2010 to July 2023, 257 patients with AA who consecutively underwent allo-HSCT were screened. Inclusion criteria included: AA diagnosis according to established criteria (6); and completion of allo-HSCT during the study period. The exclusion criteria included patients who died before engraftment (n=6), those with primary engraftment failure (n=1), or patients not evaluated for PTR within one month post-HSCT (n=35). Ultimately, 215 patients were enrolled. This study was approved by the Institutional Review Board of our hospital (IIT2021011-EC-1) and complied with the Declaration of Helsinki. Written informed consent was obtained from all participants or their legal guardians.

### 2.2 Conditioning regimen and transplantation procedure

Our institutional transplantation protocol followed established procedures as previously reported (7) and incorporated insights from haploidentical donor HSCT (HID-HSCT) protocols in China (8, 9). The regimen included: fludarabine (FLU) 150mg/m^2^ IV in divided doses on days -6 to -2, cyclophosphamide (CY) 80 or 150 mg/kg IV in a divided dose on days -5 to -2, and rATG (Thymoglobulin^®^, Genzyme, Cambridge, MA) 12.5mg/kg or porcine antilymphocyte globulin (pALG) (Anti-lymphocyte Immunoglobulin^®^, Wuhan Institute of Biological Products Co., Ltd., China) 100 or 125mg/kg IV in divided doses on days -5 to -2. For patients undergoing HID-HSCT or with transfusion-dependent AA, busulfan (BU) 6.4 mg/kg IV, divided over days –7 to –6, was added. Prophylaxis for acute GVHD (aGVHD), infection prevention, and surveillance followed prior protocols (7).

### 2.3 Definitions

Days of neutrophil and platelet engraftment (10), aGVHD (11), chronic GVHD (12), graft failure (GF) (13), and primary cause of death (COD) (14) were defined based on previously reported criteria. All patients received single-donor apheresis platelet products. The 12-hour corrected count increment (CCI) was calculated between 8 and16 hours post-transfusion (typically measured at 8 AM following transfusions administered between 4:00–5:00 PM or 11:00 PM–12:00 AM) using the standard formula:


$$\begin{array}{c} \text{CCI}=(\text{post}-\text{transfusion count}(\text{μL})\\ -\text{pre}-\text{transfusion count}(\text{μL}))\\ \times \text{body surface area}({\text{m}}^{2})/2.5(\times 10^{11}) \end{array}$$

where the denominator (2.5 × 10¹¹) represents the average platelet dose administered at our center. PTR was defined as a CCI<5 × 10^9^/L on two sequential occasions (5). Transplant-related mortality (TRM) was defined as death without GF. Transplant failure after HSCT was defined as death or GF, whichever occurred first. Failure-free survival (FFS) was defined as the time from the Day +30 post-HSCT to treatment failure or last follow-up. Overall survival (OS) was defined as the time from the Day +30 post-HSCT to death or last follow-up.

### 2.4 Statistical analysis

This study aimed to compare OS between AA patients with and without PTR within one month after allo-HSCT.

All patients attended outpatient follow-up visits or were contacted by telephone. The final follow-up date was July 2023. Continuous and categorical variables were compared using the Mann–Whitney U test and chi-square test, respectively. When cell counts were ≤ 5, Fisher’s exact test was used. The median follow-up of surviving patients was calculated using the reverse Kaplan–Meier method. The cumulative incidences (CIs) of GVHD and TRM were calculated using the competing risk model and compared using the Gray’s test. Death or graft failure was considered a competing event for GVHD. The probabilities of OS and FFS were calculated using the Kaplan–Meier method, and differences were assessed using the log–rank test. Variables with P-values ≤0.1 in univariate analysis were entered into multivariate models to identify factors affecting survival. Variables including donor type, patient age, interval from diagnosis to transplantation, and ferritin levels before transplantation were used as covariates in propensity score matching (PSM). Patients in the PTR group were matched to those in the non-PTR group using 1:4 nearest neighbor matching with a caliper width of 0.2. R (version 4.0.5), GraphPad Prism (version 5), and SPSS (version 25.0) were used for statistical analyses. Figures were generated using GraphPad Prism 5. All P-values were two-sided, and results were considered statistically significant at *P* < 0.05.

## 3 Results

### 3.1 Characteristics of patients

The study cohort included 215 AA patients undergoing allogeneic HSCT, among whom 24 (11.7%) developed platelet transfusion refractoriness (PTR) within the first post-transplant month (**Supplementary Table 1**). Propensity score matching (PSM) yielded well-balanced cohorts of 24 PTR and 96 non-PTR patients (**Table 1**). Key clinical characteristics, including donor age and sex, patient age, interval from diagnosis to transplantation, and ferritin levels before allo-HSCT were balanced after PSM. Notably, HID accounted for more than half of the patients in each cohort (14 [PTR] vs. 50 [non-PTR]). The median age of patients in the PTR group was 37.1 years (range: 12.3–57.7) versus 31.5 years (range: 7–56.8) in the non-PTR group (*P* = 0.401), while the median age of donors was 35.7 years (range: 11–52.3) versus 34.7 years (8.1–62.4), respectively (*P* = 0.88). No significant differences were observed between groups in pre-HSCT PTR, diagnosis, donor–patient sex match, blood types of donors to recipients, mononuclear cells, and CD34+ cells infused. Follow-up duration was comparable between surviving patients (PTR: 31 months vs. non-PTR: 34 months; *P* = 0.8).

**Table 1:** Characteristics of patients with acquired aplastic anemia after PSM.

Variables	With PTR (n=24)	Without PTR (n=96)	P value
Donor type, no. (%)			
MSD	10 (41.67)	44 (45.83)	0.7
HID	14 (58.33)	50 (52.08)	
MUD	0 (0.00)	2 (2.08)	
Patient age, years, median (range)	37.1 (12.3-57.7)	31.5 (7-56.8)	0.401
Patient gender (male), no. (%)	13 (54.17)	50 (52.08)	1
Donor age, years, median (range)	35.7 (11-52.3)	34.7 (8.1-62.4)	0.88
Donor gender (male), no. (%)	15 (62.50)	59 (61.46)	1
Diagnosis, no. (%)			
severe aplastic anemia	16 (66.67)	57 (59.38)	0.514
very severe aplastic anemia	5 (20.83)	29 (30.21)	
non-severe aplastic anemia	1 (4.17)	7 (7.29)	
AA-PNH	2 (8.33)	3 (3.12)	
Pregnancy history, no. (%)			
Yes	9 (37.50)	24 (25.00)	0.231
No	2 (8.33)	21 (21.88)	
Not applicable	13 (54.17)	51 (53.12)	
Presence of PNH clones, no. (%)	5 (20.83)	37 (19.37)	1
Ferritin pre-HSCT, ng/ml, median (range)	2530.5 (29.3-4394)	1147.4 (104.3-13095)	0.07
PTR pre-HSCT, no. (%)	9 (37.50)	21 (21.88)	0.188
Interval from diagnosis to transplant, moths, median (range)	6.8 (1.6-324.6)	4.6 (0.7-1415.8)	0.43
Donor-patient sex match, no. (%)			
Female to Male	8 (33.33)	21 (21.88)	0.554
Male to Female	8 (33.33)	30 (31.25)	
Male to Male	6 (25.00)	29 (30.21)	
Female to Female	2 (8.33)	16 (16.67)	
ABO matching, no. (%)			
Matched	14 (58.33)	50 (52.08)	0.291
Major mismatched	7 (29.17)	17 (17.71)	
Minor mismatched	2 (8.33)	16 (16.67)	
Major and minor mismatched	1 (4.17)	13 (13.54)	
Mononuclear cells infused, ×10^8^/kg, median (range)	10 (8-25.5)	10.1 (4.5-31.4)	0.458
CD34+ cells infused, ×10^6^/kg, median (range)	2.7 (1.7-10.4)	1.5 (3-9.1)	0.234
Follow-up of alive patients, moths, median (range)	31 (6.7-102.2)	34.0 (2-111.3)	0.8

PSM, propensity score matching; PTR, platelet transfusion refractoriness; MSD, matched-sibling donor; HID, haploidentical donor; MUD, matched unrelated donor; AA-PNH, aplastic anemia-paroxysmal nocturnal hemoglobinuria; PNH, paroxysmal nocturnal hemoglobinuria.

### 3.2 Clinical outcomes

**Figure 1:**
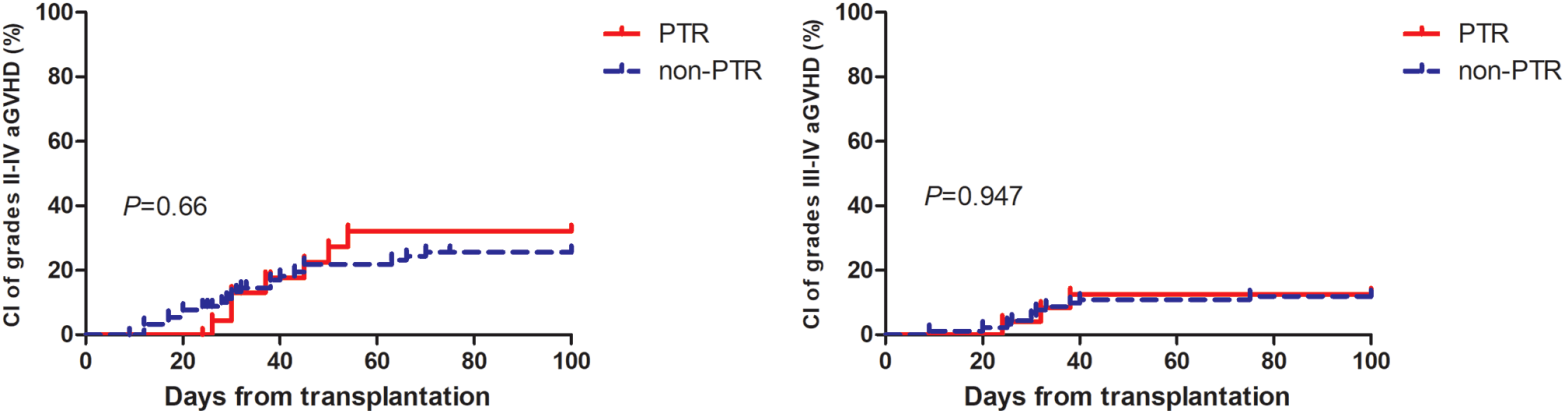
Cumulative incidence of grade II to IV aGvHD and III to IV aGvHD among AA patients with PTR were similar to patient without PTR.

**Table 2** summarizes the major clinical outcomes of patients after PSM. The CI of 100-day grade II-IV aGVHD was 32.2% (95% confidence interval [CI], 9.1–58.5) in the PTR group and 25.6% (95% CI, 12.6–41.0) (*P* = 0.66) in the non-PTR group; the CI of 100-day grade III–IV aGVHD was 12.5% (95% CI, 0.2–51.2) versus 12% (95% CI, 2.2–37.8) (*P* = 0.957), respectively (**Figure 1**). No differences were observed in terms of bloodstream infection, virus infection, and engraftment between the two groups. Notably, transfusion requirements were significantly higher in the PTR group: red blood cells: median 13.5 units (PTR) vs. 8 units (non-PTR; *P* = 0.003); platelets: median 10.5 units vs. 5 units (*P* < 0.001). Regarding TRM, the CI of 1-year TRM in the PTR group was 33.3% (95% CI, 11.0–57.9) compared to 16.9% (95% CI, 5.5–33.7) in the non-PTR group (*P* = 0.106).

**Table 2:** Major clinical outcomes of patients after PSM.

Variables	Patients with PTR (N=24)	Patients without PTR (N=96)	P value
Bloodstream infections, no. (%)	5 (20.83)	23 (23.96)	0.957
CI of 100-day of II-IV aGvHD (%) (95% CI)	32.2 (9.1-58.5)	25.6 (12.6-41)	0.660
CI of 100-day of III-IV aGvHD (%) (95% CI)	12.5 (0.2-51.2)	12 (2.2-37.8)	0.957
Neutrophil engraftment, days, median (range)	13.5 (10-23)	12 (10-21)	0.3
Platelet engraftment, days, median (range)	14 (10-27)	13.5 (9-28)	0.996
Percentage of platelet engraftment at day 28	17 (70.8)	80 (86.9)	0.243
CMV reactivation, no. (%)	9 (37.50)	43 (44.79)	0.679
EBV reactivation, no. (%)	23 (95.83)	84 (87.50)	0.419
RBC transfusions post-HSCT, units, median (range)	13.5 (1.5-73)	8 (0-128)	0.03
PLT transfusions post-HSCT, units, median (range)	10.5 (3-68)	5 (2-101)	<0.001

PSM, propensity score matching; PTR, platelet transfusion refractoriness; CI, cumulative incidence; aGvHD, acute graft versus host disease; CMV, cytomegalovirus; EBV, Epstein-Barr virus; RBC, red blood cell; PLT, platelet.

### 3.3 Survival

**Figure 2:**
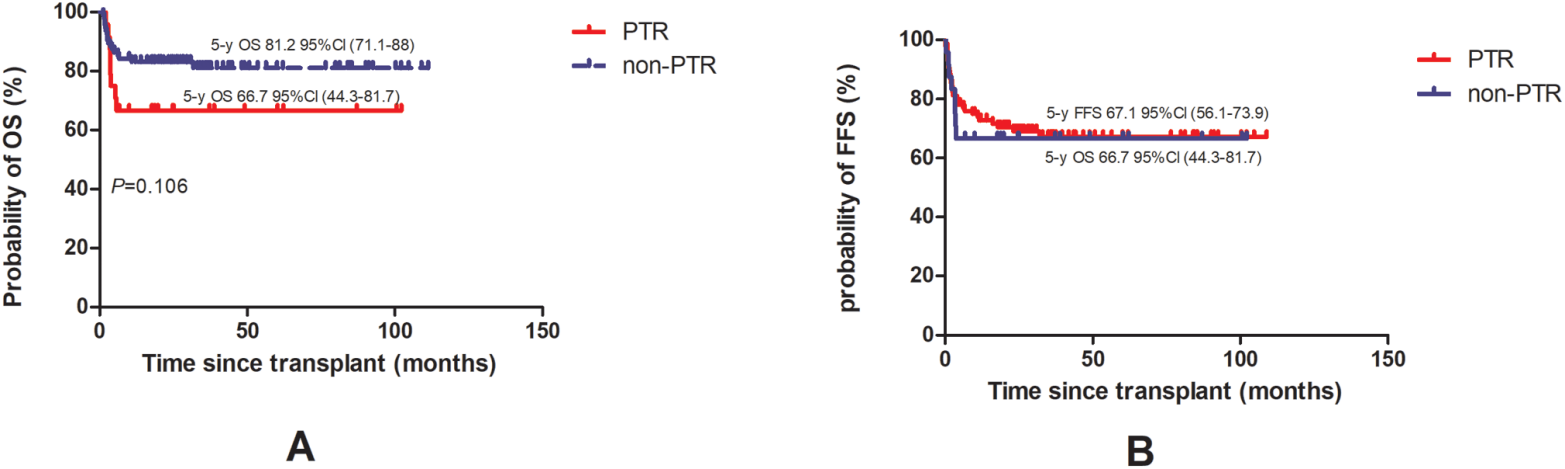
Overall survival [OS, **(A)**] and failure-free survival [FFS, **(B)**] of AA patients with PTR were compared to patient without PTR.

After PSM, the probability of 3-year OS in the PTR group was numerically lower, 66.7% (95% CI, 44.3–81.7) versus 81.2% (95% CI, 71.1–88) in the non-PTR group. However, the difference was not statistically significant (*P* = 0.106) while the probability of 3-year FFS was 66.7% (95% CI, 44.3–81.7) versus 67.1% (95% CI, 56.1–73.9) (*P* = 0.083) (**Figure 2**).

In multivariate analysis, as shown in **Table 3**, HID and patient age were independent risk factors for OS. More patients died in the PTR group (n=8) compared to those in the non-PTR group (n=15) (*P* = 0.049) while distribution of COD were similar between the two groups (**Table 4**, *P* = 0.658). The PTR group demonstrated significantly higher overall mortality (n=8 vs. n=15, *P* = 0.049). A GVHD was the predominant cause of death in both cohorts and no fatal bleeding events occurred in either group.

**Table 3:** Univariate and multivariate analysis of overall survival after PSM.

Variables	Overall survival			
	Univariate analysis		Multivariate analysis	
	HR (95%CI)	P value	HR (95%CI)	P value
Donor source				
HID vs MSD	2.34 (0.97-5.66)	0.058	2.74 (1.04 - 7.08)	0.041
MUD vs MSD	5.79 (0.71-47.34)	0.102	8.09 (0.82 - 79.65)	0.073
Patient gender (female vs male)	1.1 (0.5-2.42)	0.808	–	–
Patient age	1.06 (1.02-1.1)	0.003	1.06 (1.02 - 1.1)	0.001
Donor gender (female vs male)	0.72 (0.31-1.67)	0.445	–	–
Interval from diagnosis to transplantation	1 (1-1.01)	0.335	–	–
Ferritin preHSCT	1 (1-1)	0.805	–	–
pre-transplantation PTR (with vs without)	1.03 (0.47-2.24)	0.951	–	–
Presence of PHN (with vs without)	0.95 (0.36-2.54)	0.925	–	–
Diagnosis				
VSAA vs SAA	0.7 (0.28-1.79)	0.46	–	–
NSAA vs SAA	0 (0-Inf)	0.998		
AA-PNH vs SAA	2.07 (0.48-9)	0.332		
Donor-patient sex match				
Male to Female vs Female to Male	1.12 (0.39-3.22)	0.837	–	–
Male to Male vs Female to Male	1.12 (0.39-3.22)	0.836		
Female to Female vs Female to Male	0.84 (0.21-3.34)	0.8		
ABO matching				
Major mismatched vs Matched	1.3 (0.45-3.74)	0.628	1.4 (0.47 - 4.17)	0.548
Minor mismatched vs Matched	1.44 (0.46-4.51)	0.536	1.52 (0.44 - 5.28)	0.509
Major and minor mismatched vs Matched	2.33 (0.81-6.7)	0.117	2.53 (0.83 - 7.71)	0.102
Donor age	1.01 (0.98-1.04)	0.536	–	–
Amount of MNC	1 (0.93-1.09)	0.91	–	–
Amount of CD34+ cells	1.16 (0.93-1.45)	0.198	1 (0.79 - 1.26)	1
post-transplantation PTR (with vs without)	0.51 (0.22-1.17)	0.113	0.49 (0.19 - 1.24)	0.132

PSM, propensity score matching; HR, hazard ratio; HID, haploidentical donor; MSD, matched-sibling donor; MUD, matched unrelated donor; CI, confidence interval; PTR, platelet transfusion refractoriness; PNH, paroxysmal nocturnal hemoglobinuria; VSAA, very severe aplastic anemia; SAA, severe aplastic anemia; NSAA, non-severe aplastic anemia; AA-PNH, aplastic anemia-paroxysmal nocturnal hemoglobinuria; MNC, mononuclear cells; PTR, platelet transfusion refractoriness.

**Table 4:** Primary causes of death among patients after PSM.

COD	Patients with PTR (N=8) (%)	Patients without PTR (N=15) (%)	P value
aGVHD	5 (62.50)	9 (60.00)	0.658
Accident	1 (12.50)	3 (20.00)	
Infection	1 (12.50)	0 (0.00)	
Graft failure	0 (0.00)	1 (6.67)	
cGVHD	1 (12.50)	1 (6.67)	
TMA	0 (0.00)	1 (6.67)	

PSM, propensity score matching; COD, cause of death; PTR, platelet transfusion refractoriness; aGvHD, acute graft versus host disease; cGvHD, chronic graft versus host disease; TMA, thrombotic microangiopathy. P value here means no difference in distribution of COD between the two groups.

## 4 Discussion

To our knowledge, this represents the first study to report the incidence of PTR within the critical one-month period following HSCT in AA patients. Our data demonstrate a PTR incidence of 11.7%, which contrasts markedly with the 59.6% incidence reported by Solves et al. (15) in a mixed cohort (peripheral blood and cord blood transplants) and our own findings of 34.9% PTR incidence in myelodysplastic syndrome/myeloproliferative neoplasms (16). This lower incidence rate may be an effect of high-dose ATG universally used in conditioning regimens for AA, regardless of donor type. Supporting this, Gao et al. reported, 21 (72.40%) of the 29 PTR patients with SAA treated with ATG showed a response, while 13 (44.8%) patients had a rapid response after the first dose of ATG administration (5).

While multiple studies have associated post-HSCT platelet transfusion refractoriness (PTR) with inferior survival outcomes (3, 15, 17), our analysis of AA patients revealed no significant survival difference. This discrepancy may be explained by several key factors. First, different disease types were enrolled. We analyzed only AA patients undergoing allo-HSCT, whereas other studies included various hematological diseases. Second, different time points were studied. In this study, we focused on the period from stem cell infusion to one-month post-HSCT to reduce the effect of viral reactivation and aGVHD occurring after one month on PTR. Similarly, Tanoue et al. demonstrated that only PTR occurring 31–45 days after cord blood transplantation, rather than PTR before or after HSCT within one month, was significantly associated with inferior survival (4). Third, combined therapeutic approaches may reduce the hazards of PTR. Currently, thrombopoietin receptor ( (18–21), HLA-matched platelets, and double platelet transfusions can overcome the risk of PTR, and once engraftment is achieved within one-month post-allo-HSCT, the adverse effect of PTR may not persist.

Consistent with established literature, advanced patient age remains a significant prognostic factor for allo-HSCT outcomes in SAA patients across all donor types. Gupta et al. reported markedly increased mortality risks in older recipients of HLA-matched sibling transplants, with relative risks of 2.70 (*P* < 0.0001) for patients >40 years and 1.69 (*P* < 0.001) for those aged 20–40 years compared to younger patients (<20 years) (22). This age-dependent survival pattern has been consistently observed in matched unrelated donor (MUD) transplant settings as well (23, 24). Notably, our analysis identified HID-HSCT as an additional independent risk factor for overall survival. This finding likely reflects the selective use of HID-HSCT as salvage therapy at our institution, primarily for patients with both prolonged diagnosis-to-transplant intervals and extensive pre-transfusion histories that may collectively contribute to the poorer outcomes observed in this subgroup.

This study has several important limitations that should be acknowledged. First, our sample size was small which may undermine statistical power and selection bias was unavoidable. For example, patients who died before engraftment may have been more likely to experience PTR; however, we did not analyze this population in our study to avoid blowing up the effect of PTR instead of infections or conditioning toxicities. Second, the reasons for and risk factors of PTR were not explored in detail in our study. In recent years at our center, for patients proceeding to or undergoing allo-HSCT, we have applied tests including anti-class I human leukocyte antigens, anti-human platelet antigens, anti-membrane glycoproteins, and anti-CD36 antibodies to screen for the causes of PTR. Of note, HLA antibodies derived from donor cells post-HSCT have been reported (25). However, non-immunologic factors such as infection, drugs, and increased consumption caused by fever post-allo-HSCT remain the major causes in clinical practice (26, 27). Third, our results should be confirmed in prospective, multi-centers studies.

Through PSM analysis, we demonstrate that early-onset PTR, within one month post-HSCT, may not adversely affect survival outcomes in AA patients. Our findings suggest that incorporation of rATG or pALG in conditioning regimens may mitigate PTR incidence while patient age and HID status remain critical prognostic factors for overall survival. The retrospective design and moderate sample size constrain definitive conclusions. These observations warrant validation through prospective multicenter studies with larger patient cohorts and standardized PTR assessment protocols. This study provides important preliminary evidence that early post-transplant PTR may represent a manageable complication in AA patients receiving modern transplant protocols.

## Data Availability

The raw data supporting the conclusions of this article will be made available by the authors, without undue reservation.
