# Supplementary material for: Platelet transfusion refractoriness within one month post-hematopoietic stem cell transplantation does not impair survival in aplastic anemia patients after engraftment: a propensity score-matched analysis
Source: Front Immunol. 2025 Jul 24;16:1623004. doi: 10.3389/fimmu.2025.1623004 (PMC12328195; doi:10.3389/fimmu.2025.1623004)
Supplement: Supplementary file 1 [file Table1.docx]

**Characteristics of enrolled patients with acquired aplastic anemia**

| **Variables** | **With PTR (n=24)** | **Without PTR (n=191)** | ***P* value** |
| --- | --- | --- | --- |
| Donor type, no. (%) |  |  | 0.441 |
| MSD | 10 (41.67) | 101 (52.88) |  |
| HID | 14 (58.33) | 87 (45.55) |  |
| MUD | 0 (0.00) | 3 (1.57) |  |
| Patient age, years, median (range) | 37 (12.3-57.7) | 22.9 (3.5-56.8) | 0.002 |
| Patient gender (male), no. (%) | 13 (54.17) | 114 (59.69) | 0.766 |
| Donor age, years, median (range) | 35.7 (11-52.3) | 31.3 (8.1-62.4) | 0.266 |
| Donor gender (male), no. (%) | 15 (62.50) | 109 (57.07) | 0.773 |
| Diagnosis, no. (%) |  |  | 0.299 |
| severe aplastic anemia | 16 (66.67) | 110 (57.59) |  |
| very severe aplastic anemia | 5 (20.83) | 62 (32.46) |  |
| non-severe aplastic anemia | 1 (4.17) | 14 (7.33) |  |
| AA-PNH | 2 (8.33) | 5 (2.62) |  |
| Pregnancy history, no. (%) |  |  |  |
| Yes | 9 (37.50) | 31 (16.23) | 0.023 |
| No | 2 (8.33) | 45 (23.56) |  |
| Not appliable | 13 (54.17) | 115 (60.21) |  |
| Presence of PNH clones, no. (%) | 19 (79.17) | 154 (80.63) | 1 |
| Ferritin pre-HSCT, ng/ml, median (range) | 2030.5 (29.3-4394) | 1034.45 (12.2-13095) | 0.09 |
| PTR pre-HSCT, no. (%) | 9 (37.50) | 40 (20.94) | 0.118 |
| Interval from diagnosis to transplant, moths, median (range) | 43.229 (71.787) | 35.038 (66.614) | 0.597 |
| Donor-patient sex match, no. (%) |  |  | 0.848 |
| Female to Male | 8 (33.33) | 57 (29.84) |  |
| Male to Female | 8 (33.33) | 53 (27.75) |  |
| Male to Male | 6 (25.00) | 57 (29.84) |  |
| Female to Female | 2 (8.33) | 24 (12.57) |  |
| Blood types of donors to recipients, no. (%) |  |  | 0.259 |
| Matched | 14 (58.33) | 113 (59.16) |  |
| Major mismatched | 7 (29.17) | 29 (15.18) |  |
| Minor mismatched | 2 (8.33) | 34 (17.80) |  |
| Major and minor mismatched | 1 (4.17) | 15 (7.85) |  |
| Mononuclear cells infused, ×108/kg, median (range) | 10 (8-25.5) | 9.4 (3.1-31.4) | 0.12 |
| CD34+ cells infused, ×106/kg, median (range) | 2.7 (1.7-14) | 3 (0.5-14) | 0.171 |
| Follow-up of alive patients, moths, median (range) | 31.1 (6.7-102.2) | 36.5 (2-111.3) | 0.642 |
